# Adaptation and validation of an influenza a subtyping panel for detection of H1pdm09, H3 and H5 on a high-throughput RT-qPCR system

**DOI:** 10.1038/s41598-026-45563-5

**Published:** 2026-04-20

**Authors:** Katja Giersch, Dominik Nörz, Moritz Grunwald, Hui Ting Tang, Lisa Sophie Pflüger, Susanne Pfefferle, Anne Pohlmann, Martin Beer, Martin Aepfelbacher, Timm Harder, Marc Lütgehetmann

**Affiliations:** 1 Institute of Medical Microbiology, Virology and Hygiene, University Medical Centre Hamburg-Eppendorf (UKE), Hamburg, Germany; 2 Friedrich-Loeffler-Institut Bundesforschungsinstitut für Tiergesundheit, Institut für Virusdiagnostik, Greifswald-Insel Riems, Greifswald, Germany; 3 German Center for Infection Research (DZIF), Hamburg-Lübeck-Borstel- Riems, Germany; 4 Institute of Medical Microbiology, Virology and Hygiene, University Medical Center Hamburg-Eppendorf, Martinistraße 52, D-20246 Hamburg, Germany

**Keywords:** H1N1, H3N2, H5, Infection, Real time polymerase chain reaction, Cobas 5800, Cobas 6800, Cobas 8800, Molecular diagnostics, Avian influenza, Spillover, Biological techniques, Diseases, Microbiology

## Abstract

Facing the emergence of highly pathogenic avian influenza virus (HPAIV) H5N1 clade 2.3.4.4b in birds and its circulation in dairy cattle, rapid and reliable assays to detect HPAIV infection in humans are needed in diagnostic laboratories worldwide. We adapted and evaluated the performance of a molecular influenza A subtyping assay for the detection of A(H1N1)pdm09, A(H3N2), A(H5) and a pan-influenza A target on a high-throughput, fully automated platform. Previously published target primers and probes (Panning et al., Terrier et al., and the World Health Organization) were modified for target inclusivity/exclusivity, and adapted to compatibility with the Roche cobas5800/6800/8800 system as multiplex reaction. To evaluate the analytical performance of the new subtyping assay external quality assessment (EQA) samples were used as standards and quantified by digital-PCR. Limits of detection determined by 95% probit analysis were 754.0 digital copies (dcp)/ml for A(H1N1)pdm09, 148.0 dcp/ml for A(H3N2), 156 dcp/ml for A(H5) and 45.2 dcp/ml for the influenza A pan-target. Linearity was assessed for each subtyping target over at least four log-steps (r^2^: 0.9969–0.9998). The assay showed 100% agreement with EQA samples, eluates from the Friedrich Loeffler Institut (Germany) tested positive for different influenza A subtypes, and CE-IVD manual tests using 132 clinical samples. No false positives were detected in the exclusivity set. Our new subtyping assay is fully automated, easily scalable and can be used in surveillance and routine clinical settings enabling the detection of HPAIV infections in humans and may contribute to limit potential transmission chains at an early stage. Words: 250/250.

**Supplementary Information:**

The online version contains supplementary material available at 10.1038/s41598-026-45563-5.

## Introduction

Influenza A viruses are constantly evolving and are classified into different subtypes, clades and subclades based on the two virus surface proteins hemagglutinin (H) and neuraminidase (N)^1^. Influenza A subtypes that routinely circulate in people are A(H1N1)pdm09 and A(H3N2), while influenza A viruses from birds and swine sporadically infect humans^1^. However, zoonotic infections e.g. with highly pathogenic avian influenza viruses (HPAIV) such as A(H5N1) have the potential to adapt to humans, leading to human-to-human transmission and posing pandemic threats^2–4^.

Recently HPAIV A(H5N1) clade 2.3.4.4b (B3.13 genotype) was detected in dairy cattle and raw milk in the USA^5,6^, and transmission to humans was observed, raising global health concerns^7^. Since April 2024, the Centers for Disease Control and Prevention (CDC) confirmed 70 human A(H5N1) clade 2.3.4.4b cases in the USA., two-thirds of which due to contact with HPAIV-infected dairy cows^8^. While most of these infected patients showed mild symptoms, two cases with severe symptoms have been reported in USA and Canada after contact to birds^9^. In both cases infection was related to clade 2.3.4.4b genotype D1.1 which is currently circulating in wild birds and poultry in North America^10,11^. Although, human-to-human spread was not observed, adaptive mutations to increase viral replication in human cells were observed during course infection^11^. Therefore, continuous global monitoring and rapid detection of human infections with novel influenza A viruses such as A(H5N1)2.3.4.4b, are important to facilitate prompt awareness and an effective public health response^12^. To date, several published qPCR assays for detection of influenza virus A (H5) in humans and animals are available^13–16^, however most assays rely on manual qPCR setup. Additionally, the COVID-19 pandemic has highlighted the importance of a rapid molecular pathogen detection on easily scalable and high throughput systems^17^.

In this study, we report the analytical evaluation of a laboratory-developed real-time RT-qPCR test for the detection and typing of influenza A variants including H5 using the open channel of the high-throughput Roche cobas 5800/6800/8800 systems.

## Methods

### **Setup of influenza a subtyping assay**

 The influenza A subtyping RT-qPCR assay was compiled based on previously published primer/probe sets or target regions for detecting the variants A(H1N1)pdm09^18^, A(H3N2)^19^, A(H5)^19^ and a influenza A pan-target in the M-gene^20^. Primer and probes were modified for best inclusivity, reduced oligo interaction and compatibility with the cobas 5800/6800/8800 system (Roche Diagnostics, Rotkreutz, Switzerland) as described in Table 1a **and supplementary methods**.

**Table 1:**
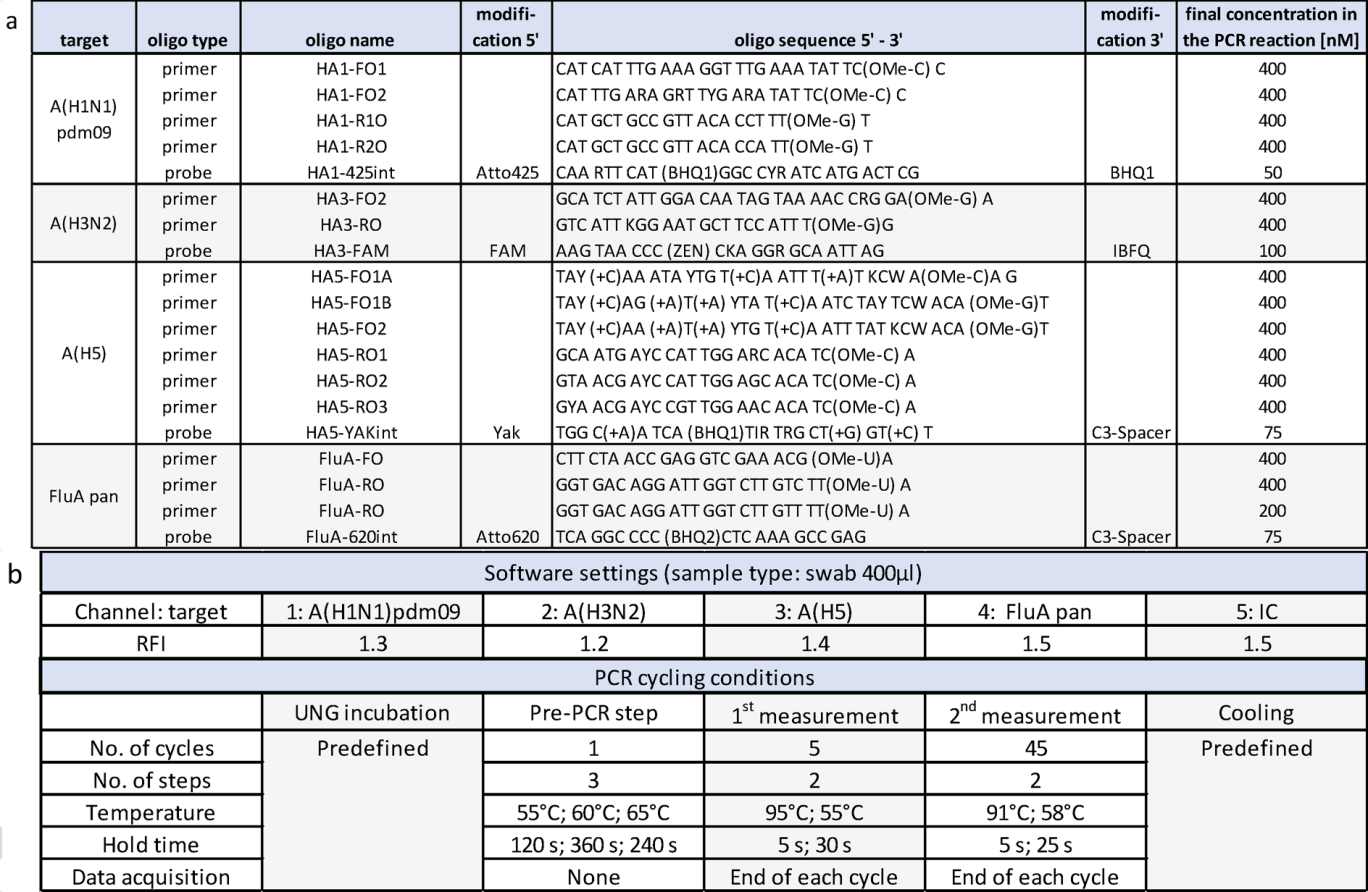
Oligo sequences of the influenza A subtyping assay. ***a***: Atto425, FAM, YAK and Atto620 are different fluorescent dyes that allow detection of each target in one of the four cobas5800/6800/8800 channels. OMe-X: 2’O-methyl-RNA bases, (+ Y): LNA bases, ZEN/IBFQ: ZEN–Iowa Black fluorescence quencher, BHQ1: Black Hole Quencher 1, I: ionsine. ***b***: Cobas omni Utility Channel software settings and run protocol. Automated result calls use relative fluorescence increase (RFI) thresholds. IC: internal control. UNG: Uracil-DNA Glycosylase, an enzyme to prevent PCR carryover contamination.

Cobas 5800/6800/8800 systems allow the implementation of in-house qPCR tests by using the open channel with the cobas omni utility channel reagent cassettes (Roche Diagnostics, Rotkreutz, Switzerland) which are compliant with European regulations (CE-IVD). Nucleic acid extraction from primary samples and RT-qPCR measurement are fully automated and are performed in a single integrated workflow, which consists of an Uracil-DNA Glycosylase (UNG) incubation to prevent PCR carryover contamination, a pre-PCR (RNA transcription) and 1 st (baseline level detection) and 2nd measurement (fluorescence detection). Four different channels are used simultaneously to detect different pathogen targets, while the fifth channel provides a spike-in RNA full-process control that is automatically added during extraction.

The preparation of the Cobas omni utility channel reagent kit (Roche Diagnostics, Rotkreutz, Switzerland) and the run protocol are described in Table 1b and in **supplementary methods.**

### In-silico inclusivity and cross-reactivity of primers and probes

In-silico inclusivity was evaluated according to data bank queries from GenBank (assessed December 2023) and by analysing between 11,686 and 20,263 sequences per target (Table 2). All A(H1N1)pdm09, A(H3N2) and A(H5N1), A(H5N2), A(H5N6) und A(H5N8) sequences retrieved up to 2023 were included. Potential cross-reactivity to other influenza A variants of the oligos used in our subtyping assay were determined by downloading and analyzing 1,052 influenza A gene sequences available on the Global Initiative on Sharing All Influenza Data (GISAID) up to May 20, 2024. Alignments to the full oligo set were performed using Geneious Prime software (version 2025.1.2).

### Evaluation of analytical performance

 Technical evaluation of the influenza A subtyping assay for swab (UTM) clinical samples was performed on a cobas 5800 according to the European Union In Vitro Diagnostics Regulation (2017/746 EU IVDR^21^. The cobas 6800 and 8800 systems are fully comparable to the cobas 5800 series sharing the same assay menu, reagent concept and user interface. To evaluate the analytical sensitivity external quality assessment (EQA) samples from INSTAND (Duesseldorf, Germany), that were positive for A(H1N1)pdm09, A(H3N2) and A(H5), were used as standards and quantified by digital-PCR using the Qiacuity (Qiagen, Hilden, Germany) digital-PCR system following the manufacturer’s instructions. The lower limit of detection (LoD) was determined by 95% probit analysis (CLSI EP17-A2^22^) in digital copies/milliliter (dcp/ml) and using serial two-fold dilutions of the standard with eight dilution steps and eight replicates.

Linearity was assessed by ten-fold serial dilution of the same influenza A virus variant standards that were used for the analytical sensitivity study (four to six dilution steps, *n* = 10 per dilution). The linear range was calculated and depicted as simple linear regression using Validation Manager software (Finbiosoft, Espoo, Finland) and GraphPad Prism 10.4.0 (Boston, USA). The goodness of fit was determined by calculating the R squared value.

The within-run and between-day precision was determined on three days and by using two high positive (ct: 20.0–29.9), two low positive (ct: 30.8–38.6) and one negative EQA sample for each virus variant in triplicates. Within-lab precision was calculated as sum of squares of precision components. Precision was calculated as standard deviation (SD) with coefficient of variation (CV %) according to ANOVA statistics using Validation Manager (Finbiosoft).

To further analyse inclusivity of the new assay, one swine (H3N2), seven HPAIV and one low pathogenic avian influenza A (LPAIV) RNA positive eluate from the German influenza A virus Reference Center (Friedrich Loeffler Institut, Greifswald – Riems, Germany) containing different subtypes, pathotypes and clades (6x H5N1, 1x H5N2 and 1x H5N8) were analysed. 40 µl of RNA eluate were added to 560 µl cobas PCR medium (Roche Diagnostics, Rotkreutz, Switzerland) and directly measured on the cobas 5800/6800/8800 platform.

Ten EQA samples from the INSTAND (Duesseldorf, Germany) influenza virus panel (#370, 2023/2024) were analysed with our new influenza A subtyping assay. The EQA samples contained patient isolates from cell culture lysates similar to A/Wisconsin/588/2019 (A(H1N1)pdm09), A/Cambodia/e0826360/2020 (A(H3N2)) and A/Whooper Swan/R65/2006 (A(H5)).

A cross-reactivity study was performed using 10 influenza B virus positive clinical samples (ct: 16.0 to 25.7) as well as 22 clinical samples containing different viruses (e.g. rhinovirus, human metapneumovirus) and 3 fungi and 32 bacterial isolates (e.g. *Escherichia coli*,* Klebsiella. pneumoniae)*. In addition, one swine (H1N2) and four LPAIV positive eluates of subtypes H1N1, H2N9, H6N1 and H8N4 from the German Influenza A Virus Reference Centre were included in the exclusivity study and measured as described above.

### Clinical evaluation

For clinical validation, 152 clinical respiratory samples positive for influenza A from routine screening were typed using our new assay on cobas 5800/6800/8800 systems and compared to three manual CE-IVD Allplex Respiratory Panel 1 A Assays from Seegene (Dusseldorf, Germany). Nucleid acids were extracted from 200 µl nasopharyngeal swab samples by using the MagNA Pure 96 DNA and Viral NA Small Volume Kit and a MagNA-Pure 96 instrument (Roche, Basel, Switzerland) according to the manufacturer´s instructions and manual qPCR was performed on a CFX96 (Bio-rad, Hercules, CA, USA). Samples that did not yield a typing result on the CE-IVD Allplex Respiratory Panel 1 A reference assay were excluded from the analysis. Pearson quantitative correlations and p values were calculated using GraphPad Prism 10.4.0. Kappa correlations were calculated using the “Quantify agreement with kappa” online calculator from GraphPad.

This work was conducted in accordance with § 12 of the Hamburg hospital law (§ 12 HmbKHG). The use of anonymized remnant diagnostic samples from patients was approved and informed consent was waived by the ethics committee of the Hamburg Medical Association (PV5626).

## Results

### Evaluation of influenza A subtyping assay performance

 In-silico inclusivity analysis (GenBank, December 2023) showed that at least 99.0% of A(H1N1)pdm09, A(H3N2) and FluA pan sequences and 97.4% of A(H5) sequences had no or one mismatch per oligo (Table 2).

**Table 2:**
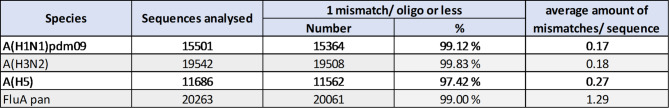
In-silico inclusivity analysis.

Overview of GenBank data bank queries from December 2023.

In-silico cross-reactivity analysis using 1,052 different influenza A gene sequences (GISAID database^23^, May 2024) showed that only certain H2 hemagglutinin sequences have a relevant risk for cross-reactivity, due to only two mismatches of the A(H5) forward and reverse primer and A(H5) probe (**suppl. Table 1**). For avian (A)H1, A(H6), and A(H8), low risks of cross-reactivity were identified with more than 5 mismatches of oligos from our subtyping assay or lack of probe binding (**suppl. Table 1**). The other variants tested showed no potential risk of cross-reactivity (**suppl. Table 1**).

**Fig. 1:**
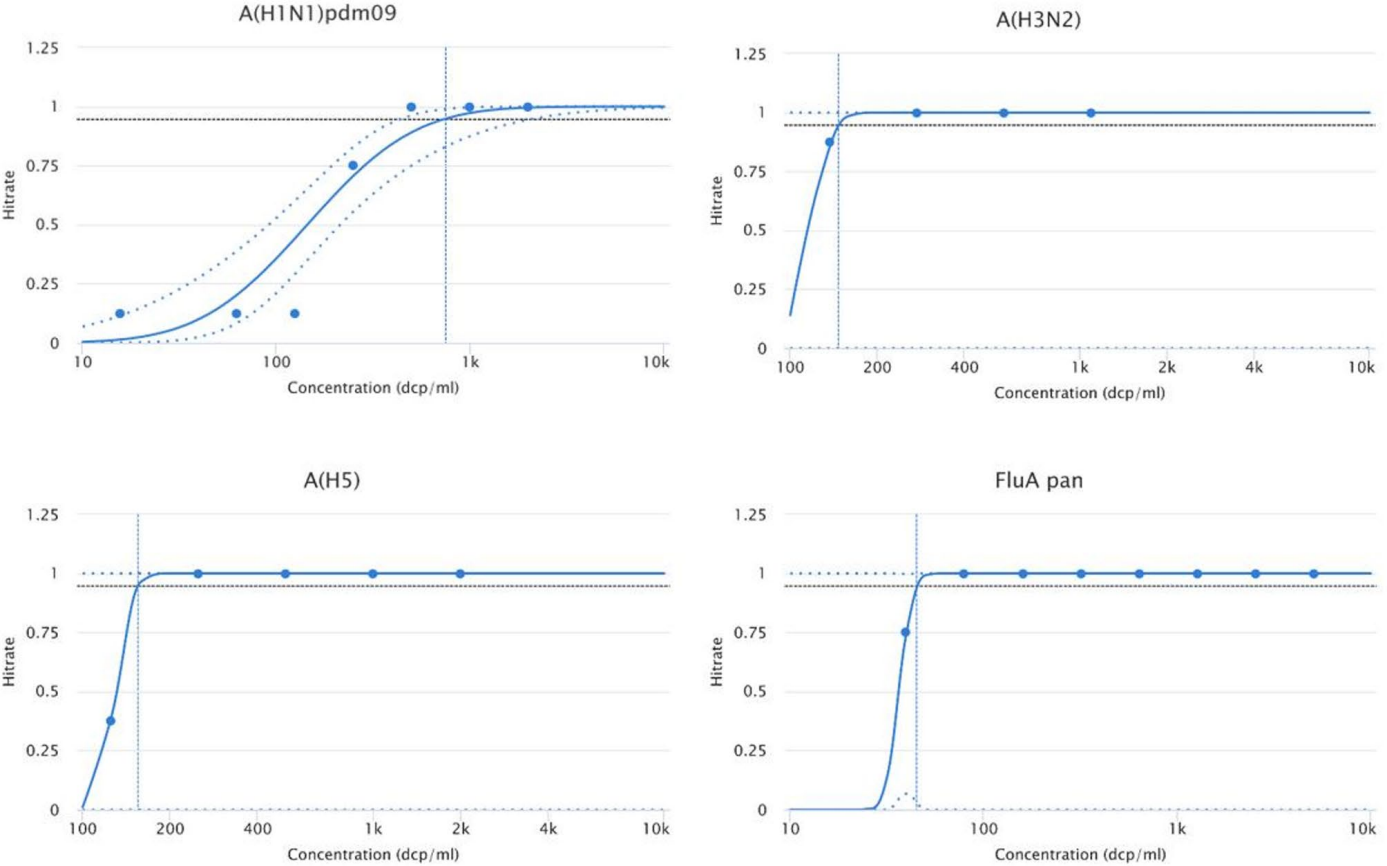
Probit curves of LoD experiment. Concentrations are shown on a logarithmic scale. Confidence intervals are indicated as dash lines. Confidence interval for A(H1N1)pdm09 are 445–2,150 dcp/ml. Confidence intervals for the other targets cannot be calculated since multiple concentrations that give both positive and negative results are required (see also hit-rates in **suppl. Table 2**).

Analytical sensitivity was assessed by using quantified EQA samples as standards in serial two-fold dilutions at eight dilution steps with eight repeats. LoDs were analysed by 95% probit analysis (CLSI EP17-A2^22^) and were 754.0 dcp/ml for A(H1N1)pdm09, 148.0 dcp/ml for A(H3N2), 156 dcp/ml for A(H5) and 45.2 dcp/ml for the FluA pan-target **(**Fig. 1**)**. The cut-off levels for each target correspond to the relative fluorescence increase (RFI) depicted in Table 1b. Hit-rates and ct values are shown in **suppl. Table 2**.

**Fig. 2:**
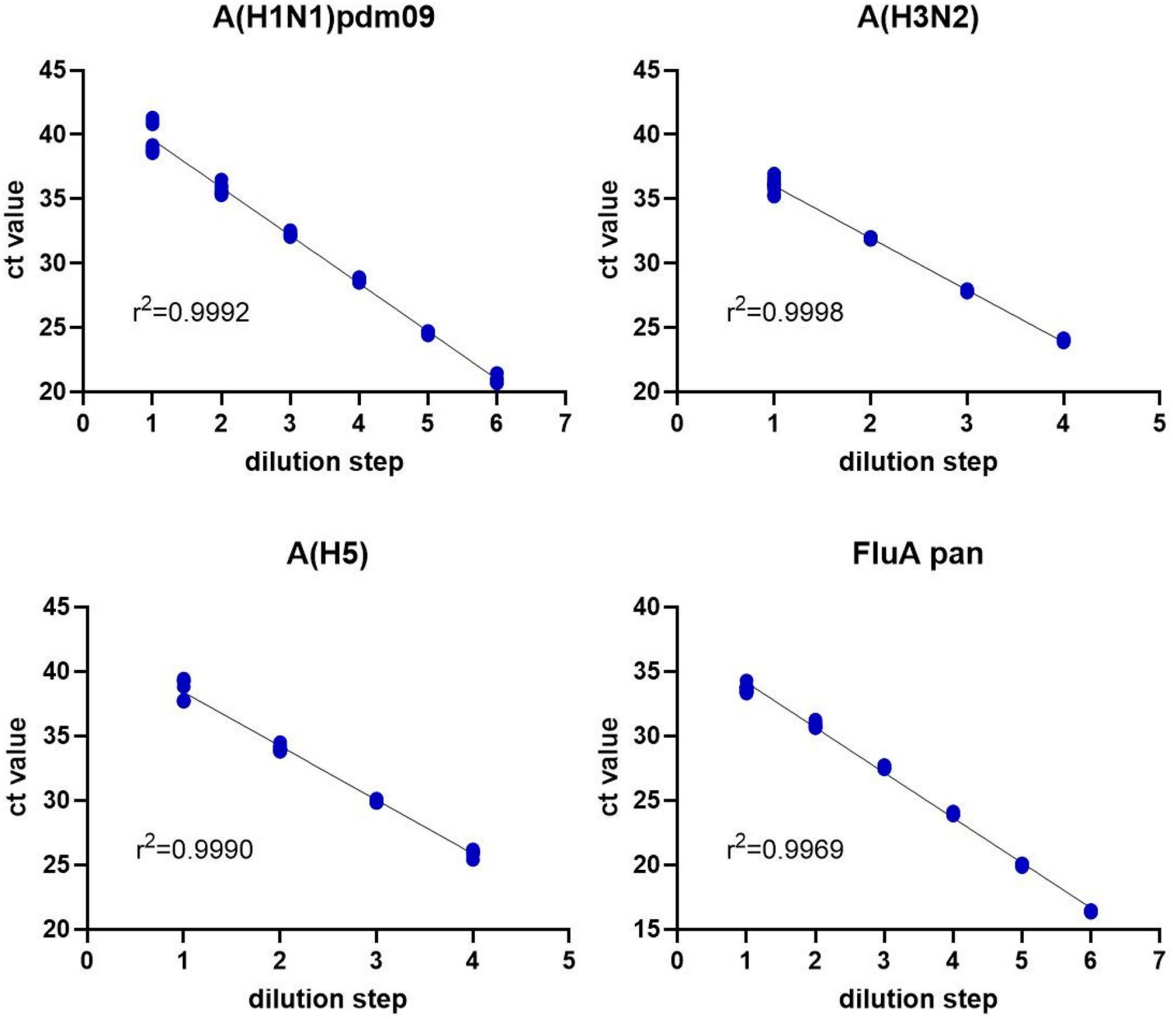
Linearity for each FluA typing target was determined using simple linear regression by serial dilution of quantified EQA samples. Goodness of fit is shown as R squared (r^2^) value in each graph.

Linearity was assessed for each subtyping target over at least four log-steps as simple linear regression. The subtyping assay showed excellent linearity for all four targets (goodness of fit shown as R squared r^2^: 0.9969–0.9998), with the following linear ranges: 20.9 ct – 39.6 ct for A(H1N1)pdm09, 24.0 ct – 36.1 ct for A(H3N2), 26.0 ct – 38.6 ct for A(H5) and 16.4 ct – 33.6 ct for FluA pan (Fig. 2).

Within-run, between-day and within-lab precision ranged between 0.03 ct and 0.26 ct for high positive samples (ct values range from 20.0 to 29.9) and between 0.04 ct and 0.68 ct for low positive samples (ct values range from 30.8 to 38.6). Negative samples for each target remained negative on all three days.

### Inclusivity and cross-reactivity

For inclusivity, seven HPAIV (6x H5N1, 1x H5N8), one LPAIV (H5N2) and one swine FluA (H3N2) positive eluate from the German influenza A virus Reference Center were analysed with our new influenza A subtyping assay. The influenza A pan-target was positive for all nine eluates and the subtypes were detected correctly (Table 3).

**Table 3:**
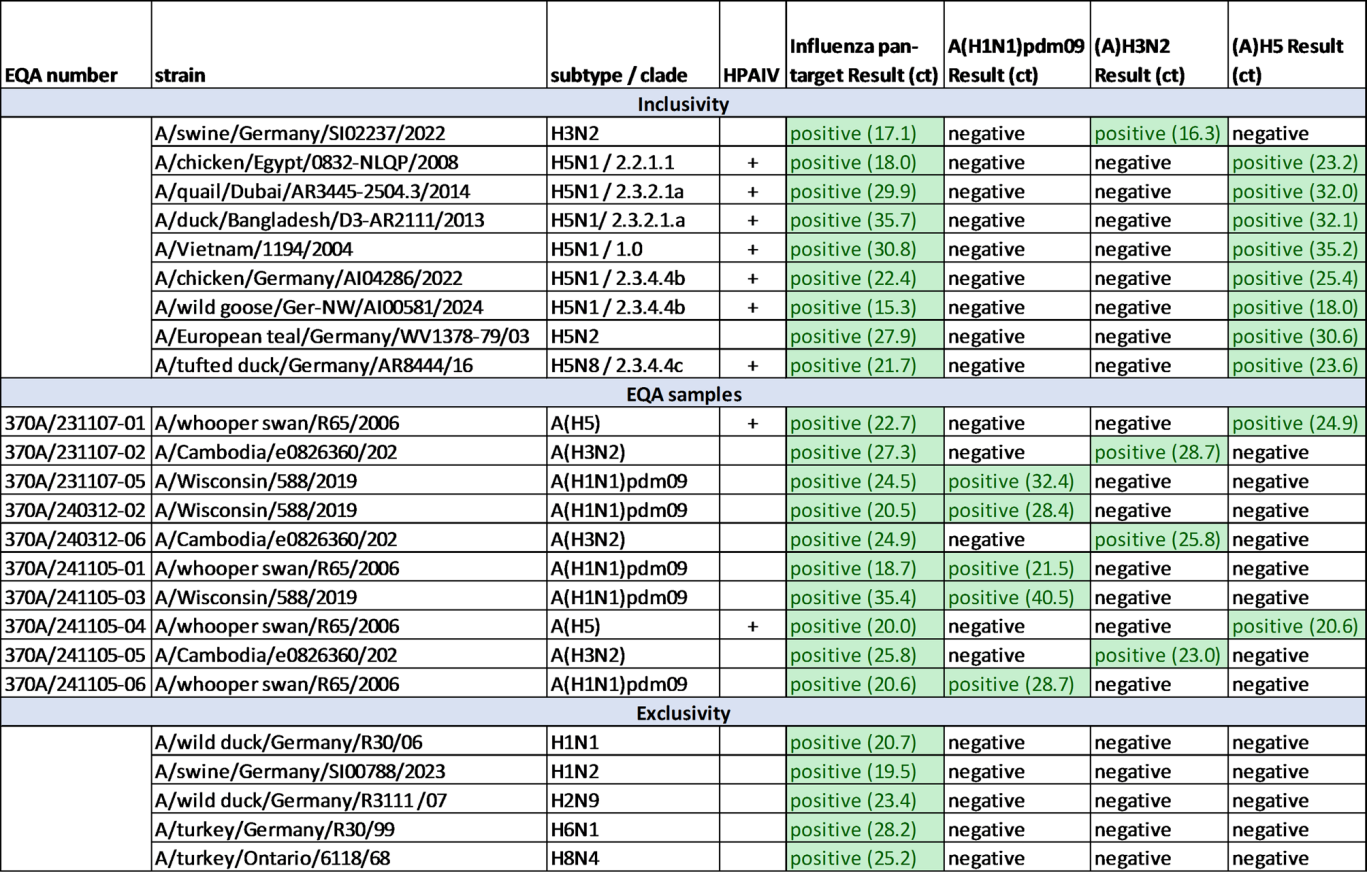
Inclusivity and exclusivity set. Eluates for the inclusivity and exclusivity set were obtained from the Friedrich Loeffler Institut (Riems, Germany) and EQA samples from INSTAND (Duesseldorf, Germany).

Additionally, ten EQA samples from the INSTAND (Duesseldorf, Germany) influenza virus panel (#370, 2023/2024) were analysed with our influenza A subtyping assay and detected correctly: 5/10 were A(H1N1)pdm09 positive, 3/10 were A(H3N2) positive and 2/10 were A(H5) positive (Table 3).

No false positives were detected in the exclusivity study, which included 22 clinical samples containing different viruses, 3 fungal isolates and 32 bacterial isolates as well as 10 influenza B virus positive samples (**suppl. Table 3**).

In addition, four different LPAIV (H1N1, H2N9, H6N1, H8N4) and one swine influenza A strain (H1N2) from the German influenza A virus Reference Center were also tested in the exclusivity panel. The eluates were positive in the pan influenza A target (ct values between 19.5 and 28.2) and negative for A(H1N1)pdm09, A(H3N2) and A(H5), confirming the specificity of our new influenza A subtyping assay (Table 3).

### Clinical validation of the influenza A subtyping assay

**Fig. 3:**
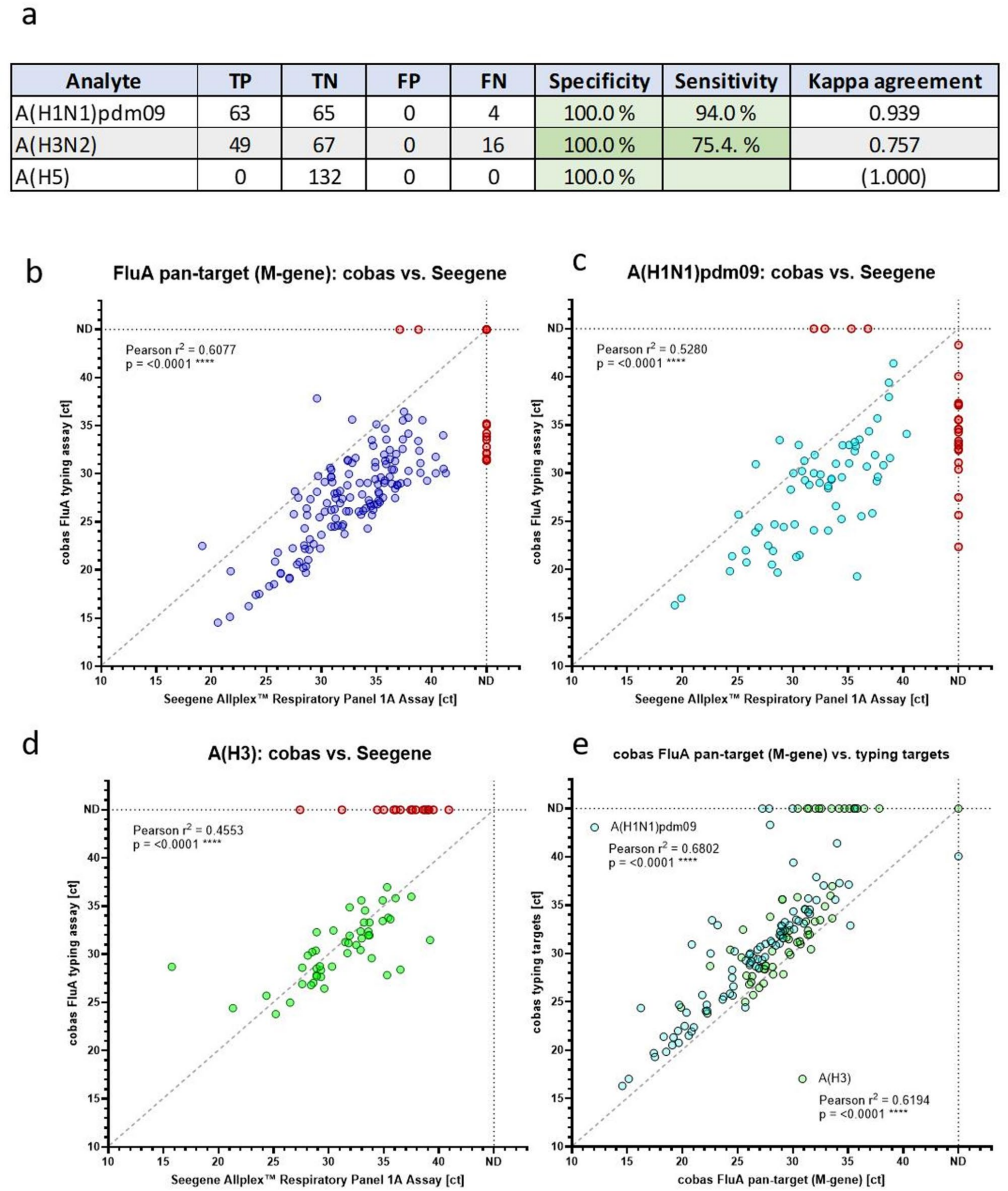
**a**) Clinical validation with 132 routine swab samples. 20 samples were excluded (no typing result in the reference assay). 20/132 were not typeable with our new assay and the remaining samples showed no contradictory typing results compared to the reference tests (CE-IVD Allplex Respiratory Panel 1 A Assays from Seegene). TP: true positive, TN: true negative, FP: false positive, FN: false negative. **b-d**) Quantitative correlation of the influenza A pan-target (**b**) A(H1N1)pdm09 (**c**) or A(H3) target (**d**) of our influenza A subtyping assay (y axis) and of the reference tests (x axis). **e**) Quantitative correlation of the influenza A pan-target (M-gene, x axis) and the HA genes of the typing targets A(H1N1)pdm09 and A(H3) (y axis) using our assay. The correlations include all 152 samples and samples that were negative in one of the two assays are shown as red dots. Dashed line: Ideal linear correlation.

Clinical respiratory samples positive for influenza A from routine screening were measured with the influenza A subtyping assay and compared to the manual CE-IVD Allplex Respiratory Panel 1 A Assay by Seegene (*n* = 152). Samples that did not show a successful typing result using the Seegene assay were excluded from the analysis (20/152; 13.2%). Of note, all of these 20 excluded samples were successfully determined as A(H1N1)pdm09 positive with our new assay (median ct: 33.8, range: 22.8–43.3). The influenza A pan-target was positive in 19/20 samples using our assay (median ct: 31.0, range: 21.0–35.9) and in 11/20 samples using the Allplex Respiratory Panel 1 A Assay by Seegene (median ct: 35.2, range: 28.8–41.1). In line, *Park et al.* recently showed that current A(H1N1)pdm09 positive subclades harbour mutations that contributed to subtyping failures using the Seegene Allplex Respiratory Panel 1 A Assay^24^. Overall, these results indicate that our new subtyping assay has a better inclusivity and sensitivity of the influenza A pan- and A(H1N1)pdm09 target than the commercially available Allplex Respiratory Panel 1 A Assay from Seegene. Using our assay, 128 out of the included 132 samples were positive for the influenza A pan-target (99.2%) and 20/132 (15.2%) samples did not show a typing result (Fig. 3a). The remaining samples were correctly typed by our assay compared to the Seegene assay (63/132 A(H1N1)pdm09 positive, 49/132 A(H3N2) positive) (Fig. 3a). The 20 samples not typed by our assay had median ct values of 34.1 (range 31.9–36.8) for A(H1N1)pdm09 (*n* = 4) and of 37.5 (range 27.4–40.9) for A(H3N2) (*n* = 16). The difficulty of subtyping samples with high ct values even with commercially available assays were also described by *Goodfellow et al.*^25^. No clinical (A)H5 positive samples were available.

Quantitative correlation of the influenza A pan-target (Fig. 3b) and the two typing targets A(H1N1)pdm09 (Fig. 3c) and A(H3N2) (Fig. 3d) of our cobas influenza A subtyping assay with the respective CE-IVD Allplex Respiratory Panel 1 A Assay from Seegene was evaluated using all 152 clinical swab samples and GraphPad Prism 10.4.0 (Boston, USA). Ct values showed good correlations (Pearson) between our new Flu A subtyping assay and the CE-IVD reference assays: r^2^ (influenza A pan) = 0.6077 (*p* < 0.0001****), r^2^ (A(H1N1)pdm09) = 0.5280 (*p* < 0.0001****) and r^2^ (A(H3N2)) = 0.4553 (*p* < 0.0001****) (Fig. 3b-d). Additionally, correlation between the cobas influenza A pan-target (M-gene) and the two typing targets A(H1N1)pdm09 and A(H3N2) showed a pearson r^2^ of 0.6802 (*p* < 0.0001****) and 0.6194 (*p* < 0.0001****), respectively (Fig. 3e).

### Clinical application of the influenza A subtyping assay

 The use of the new influenza A subtyping assay in routine diagnostics at the University Medical Center Hamburg-Eppendorf was evaluated for one year (January – December 2024). During this time 532 respiratory patient samples were detected as influenza A positive with our routine screening assay^26^(**suppl. Figure 1a**). The 508/532 samples were then applied at our new influenza A subtyping assay (24 samples had insufficient material for further subtyping), and 490/508 resulted positive with the influenza A pan-target (**suppl. Figure 1b**). Median ct values were clearly lower of samples that were detected with both assays compared to samples that were not detected as influenza A positive with the subtyping assay (ct 24.2 vs. ct 36.9 respectively) (**suppl. Figure 1b**).

Out of the 490 influenza A positive samples 92% (449 patients) were A(H1N1)pdm09 positive with a median ct of 27.5 (range 14.3–42.2), 4% (22 patients) were A(H3N2) positive with a median ct of 25.5 (range 20.4–35.6) and the remaining 4% (19 patients) did not show a typing result with our new assay (**suppl. Figure 1b**).

## Discussion

Genetic drift by unfaithful genome replication as well as genetic shifts due to re-assortment render influenza A virus genomes a constantly moving target for diagnostic approaches^27,28^. Interfaces and contacts between human and different animal species have broadened and intensified due to animal domestication, hunting, tourism and extensive urbanisation^29^. This increases the likelihood of spill-over events^2,6^. Effective surveillance and rapid detection are important to keep pace with changes in circulating influenza A strains and to identify their zoonotic potential. In particular, the emergence in humans of novel influenza A viruses from animal reservoirs requires rapid detection and characterisation due to little or no pre-existing immunity in the human population for newly introduced virus subtypes and lineages and the increased potential for global health threats^17^. In this study, we adapted and validated a RT-qPCR influenza A subtyping assay that simultaneously detects A(H1N1)pdm09, A(H3N2) and A(H5) subtypes and an influenza A pan-target in swab specimens on the fully automated, high-throughput Roche cobas 5800/6800/8800 system. In-house test development on the cobas platform is based on the cobas omni Utility Channel Kit, which includes a spike-in full process control assay. The workflow is fully automated, including bi-directional link to the laboratory information system (LIS), with a capacity of up to 1,056 samples in an eight-hour shift.

The test was validated as a secondary test to subtype known influenza A positive samples but may also be used to analyse patients with suspected influenza A(H5) infection.

Samples with a low ct value in the pan influenza A target and an inconclusive typing result can be referred to broader HA typing workflows or to whole genome sequencing^30,31^. The assay showed good analytical and clinical performance, including historical clinical and EQA samples. During one year of use in routine diagnostics, 508 influenza A -positive samples were subtyped with the new assay and 96% were successfully assigned to a subtype. Performance may degrade over time as additional mutations emerge in the respective target regions, so periodic verification of in-silico inclusivity is recommended, similar to any other in-vitro diagnostic test. It is also conceivable that the assay could be extended to include newly emerging influenza A variants such as A(H7), A(H9), A(H10).

Compared to other subtyping assays, such as virus isolation, manual lab-developed qPCR tests and commercially available CE-IVD tests, our influenza A subtyping assay produces favourable results in terms of time to result (three to four hours) and cost (approximately €10 per sample, including nucleic acid extraction; see **suppl. Table 4**). However, it should be noted that such a comparison is inherently biased by factors such as laboratory setting, number of samples analysed per flu season, availability of infrastructure, cost of materials and staff, and that the optimal solution may differ depending on local circumstances and regulatory environment. In conclusion, our new laboratory-developed, fully automated RT-qPCR assay provides rapid and easily scalable typing of current influenza A variants, including H5N1 (clade 2.3.4.4b), on a high-throughput system. The assay can be used in routine screening and outbreak management and may provide a valuable new tool to limit the transmission and impact of emerging influenza A viruses.

## Supplementary Information

Below is the link to the electronic supplementary material.

Supplementary Material 1

## Data Availability

The datasets generated and analysed during the current study are available from the corresponding author on reasonable request.
