# Supplementary material for: Adaptation and validation of an influenza a subtyping panel for detection of H1pdm09, H3 and H5 on a high-throughput RT-qPCR system
Source: Sci Rep. 2026 Apr 20;16:12888. doi: 10.1038/s41598-026-45563-5 (PMC13096109; doi:10.1038/s41598-026-45563-5)
Supplement: Supplementary file 1 — Supplementary Material 1 [file 41598_2026_45563_MOESM1_ESM.docx]

**SUPPLEMENTARY**

Katja Giersch^1#*^, Dominik Nörz ^1#^, Moritz Grunwald^1^, Hui Ting Tang^1^, Lisa Sophie Pflüger^1^, Susanne Pfefferle^1^, Anne Pohlmann^2^, Martin Beer^2^, Martin Aepfelbacher^1^, Timm Harder^2^, Marc Lütgehetmann^1,3*^

1. Institute of Medical Microbiology, Virology and Hygiene, University Medical Centre Hamburg-Eppendorf (UKE), Hamburg, Germany
2. Friedrich-Loeffler-Institut Bundesforschungsinstitut für Tiergesundheit, Institut für Virusdiagnostik, Greifswald-Insel Riems, Germany
3. German Center for Infection Research (DZIF), Hamburg-Lübeck-Borstel-Riems, Germany

# shared authorship

* corresponding author

**SUPPLEMENTARY Methods**

**Setup of influenza A subtyping assay**

**Modification of primer and probes.** Primer and probes were modified for best inclusivity, reduced oligo interaction and compatibility with the cobas 5800/6800/8800 system (Roche Diagnostics, Rotkreutz, Switzerland). To further reduce potential primer dimer formation 2’-O-methyl RNA bases were introduced at the 3’-region and locked nuclear acids (LNA; +) were added to optimize melting temperature and binding stability (**table 1a)**. Probes were internally quenched to achieve the best signal-to-noise ratio (**table 1a)**. Probe dyes were selected according to compatibility with the cobas fluorescence filter sets, and a build-in automatic dynamic colour compensation in the cobas IVD software eliminates cross talk between the channels. Primers and probes were custom-made by Ella Biotech (Fuerstenfeldbruck, Germany) and Integrated DNA Technologies (Coralville IA, USA).

**Preparation of the Cobas omni utility channel reagent kit (Roche Diagnostics, Rotkreutz, Switzerland) and run protocol.** Cobas omni utility channel reagent kit (Roche Diagnostics, Rotkreutz, Switzerland) was prepared according to manufacturer’s instructions. Briefly, primer stock (500 µM concentration) and probe stock solutions (100 µM concentration) were added to 10 ml MMX-R2 master mix, which includes a polymerase with reverse transcriptase (RT) activity. Final concentrations in the PCR reaction are depicted in **table 1a.**

Each target cut-off is described as relative fluorescence intensity (RFI) (**table 1b**), which is also used for automatic result (positive/negative) calling by the cobas omni channel software. The mode for primary liquid handling in the system was set to “swab” and the extraction volume was 400µl. The Roche generic run protocol remained unchanged to enable run compatibility with the IVD assay. The pre-PCR steps includes the RT reaction (see also **table 1b** for all settings used in this study).

**SUPPLEMENTARY Results**

**Suppl. table 1.** In-silico cross reactivity of primers and probes with other influenza A species.

| Succesfully mapped (minimum number of mismatches) | | | | |
| --- | --- | --- | --- | --- |
| **influenza A species** | **forward (fwd)** | **reverse (rev)** | **probe** | **concern** |
| avian A(H1) | A(H1)-fwd (3) | A(H5)-rev (0) | no mismatch | Low |
| A(H2) | A(H5)-fwd (2) | A(H5)-rev (2) | A(H5)-probe (2) | Relevant A(H5) |
| A(H4) | A(H3)-fwd (2) | A(H3)-rev (>5) | no mismatch | no concern |
| A(H6) | A(H1)-fwd (2) | A(H5)-rev (5) | A(H3)-probe (>5) | Low |
| A(H7) | A(H1)-fwd (>5) | no mismatch | no mismatch | no concern |
| A(H8) | A(H1)-fwd (>10) | A(H3)-rev (>5) | A(H3)-probe (>5) | Low |
| A(H9) | no mismatch | A(H5)-rev (>5) | no mismatch | no concern |
| A(H10) | A(H3)-fwd (4) | no mismatch | no mismatch | no concern |
| A(H11) | A(H5)-fwd (>5) | A(H5)-rev (>5) | no mismatch | no concern |
| A(H12) | no mismatch | no mismatch | no mismatch | no concern |
| A(H13) | A(H5)-fwd (>5) | no mismatch | no mismatch | no concern |

*(Numbers in brackets show the minimum number of mismatches from the alignments using a total of 1,052 influenza A HA gene sequences)*

**Suppl. table 2: Hit rates (LOD)**

**Influenza A**

|  |  | | |
| --- | --- | --- | --- |
| Concentration | Positive results | Hit-rate | Average Ct |
| 39.8 copies/ml | 6 / 8 | 0.75 | 36.5 |
| 79.7 copies/ml | 8 / 8 | 1.00 | 34.5 |
| 159 copies/ml | 8 / 8 | 1.00 | 33.6 |
| 319 copies/ml | 8 / 8 | 1.00 | 32.5 |
| 638 copies/ml | 8 / 8 | 1.00 | 31.5 |
| 1,280 copies/ml | 8 / 8 | 1.00 | 30.5 |
| 2,550 copies/ml | 8 / 8 | 1.00 | 29.6 |
| 5,100 copies/ml | 8 / 8 | 1.00 | 28.7 |

**A(H1N1)pdm09**

|  |  | | |
| --- | --- | --- | --- |
| Concentration | Positive results | Hit-rate | Average Ct |
| 15.6 copies/ml | 1 / 8 | 0.125 | 41.1 |
| 31.3 copies/ml | 0 / 8 | 0 | N/A |
| 62.5 copies/ml | 1 / 8 | 0.125 | 41.4 |
| 125 copies/ml | 1 / 8 | 0.125 | 41.7 |
| 250 copies/ml | 6 / 8 | 0.75 | 40.4 |
| 500 copies/ml | 8 / 8 | 1.00 | 39.4 |
| 1,000 copies/ml | 8 / 8 | 1.00 | 38.5 |
| 2,000 copies/ml | 8 / 8 | 1.00 | 37.5 |

**A(H3N2)**

|  |  | | |
| --- | --- | --- | --- |
| Concentration | Positive results | Hit-rate | Average Ct |
| 8.59 copies/ml | 0 / 8 | 0 | N/A |
| 17.2 copies/ml | 0 / 8 | 0 | N/A |
| 34.4 copies/ml | 0 / 8 | 0 | N/A |
| 68.8 copies/ml | 0 / 8 | 0 | N/A |
| 138 copies/ml | 7 / 8 | 0.875 | 38.8 |
| 275 copies/ml | 8 / 8 | 1.00 | 36.0 |
| 550 copies/ml | 8 / 8 | 1.00 | 34.4 |
| 1,100 copies/ml | 8 / 8 | 1.00 | 33.1 |

**A(H5)**

|  |  | | |
| --- | --- | --- | --- |
| Concentration | Positive results | Hit-rate | Average Ct |
| 15.6 copies/ml | 0 / 8 | 0 | N/A |
| 31.3 copies/ml | 0 / 8 | 0 | N/A |
| 62.5 copies/ml | 0 / 8 | 0 | N/A |
| 125 copies/ml | 3 / 8 | 0.375 | 38.6 |
| 250 copies/ml | 8 / 8 | 1.00 | 37.2 |
| 500 copies/ml | 8 / 8 | 1.00 | 35.5 |
| 1,000 copies/ml | 8 / 8 | 1.00 | 34.1 |
| 2,000 copies/ml | 8 / 8 | 1.00 | 32.8 |

*(Hit-rates and average ct values obtained from the LoD experiment for each dilution step of the four targets of the influenza A subtyping assay.)*

**Suppl. table 3. Cross-reactivity study.**

| **Pathogen** | **Results** | | | |
| --- | --- | --- | --- | --- |
|  | influenza A pan | A(H1pdm09) | A(H3N2) | A(H5) |
| **Viruses** |  |  |  |  |
| Adenovirus | neg (no ct) | neg (no ct) | neg (no ct) | neg (no ct) |
| Adenovirus | neg (no ct) | neg (no ct) | neg (no ct) | neg (no ct) |
| BK virus | neg (no ct) | neg (no ct) | neg (no ct) | neg (no ct) |
| BK virus | neg (no ct) | neg (no ct) | neg (no ct) | neg (no ct) |
| Bocavirus | neg (no ct) | neg (no ct) | neg (no ct) | neg (no ct) |
| Cytomegalovirus | neg (no ct) | neg (no ct) | neg (no ct) | neg (no ct) |
| Enterovirus | neg (no ct) | neg (no ct) | neg (no ct) | neg (no ct) |
| Epstein–Barr virus | neg (no ct) | neg (no ct) | neg (no ct) | neg (no ct) |
| Epstein–Barr virus | neg (no ct) | neg (no ct) | neg (no ct) | neg (no ct) |
| Herpes simplex virus 1 | neg (no ct) | neg (no ct) | neg (no ct) | neg (no ct) |
| Herpes simplex virus 1 | neg (no ct) | neg (no ct) | neg (no ct) | neg (no ct) |
| Herpes simplex virus 2 | neg (no ct) | neg (no ct) | neg (no ct) | neg (no ct) |
| Herpes simplex virus 2 | neg (no ct) | neg (no ct) | neg (no ct) | neg (no ct) |
| Human coronavirus | neg (no ct) | neg (no ct) | neg (no ct) | neg (no ct) |
| Human herpesvirus 6 | neg (no ct) | neg (no ct) | neg (no ct) | neg (no ct) |
| Human herpesvirus 8 | neg (no ct) | neg (no ct) | neg (no ct) | neg (no ct) |
| Human metapneumovirus | neg (no ct) | neg (no ct) | neg (no ct) | neg (no ct) |
| Human papillomavirus | neg (no ct) | neg (no ct) | neg (no ct) | neg (no ct) |
| Influenza B virus | neg (no ct) | neg (no ct) | neg (no ct) | neg (no ct) |
| Influenza B virus | neg (no ct) | neg (no ct) | neg (no ct) | neg (no ct) |
| Influenza B virus | neg (no ct) | neg (no ct) | neg (no ct) | neg (no ct) |
| Influenza B virus | neg (no ct) | neg (no ct) | neg (no ct) | neg (no ct) |
| Influenza B virus | neg (no ct) | neg (no ct) | neg (no ct) | neg (no ct) |
| Influenza B virus | neg (no ct) | neg (no ct) | neg (no ct) | neg (no ct) |
| Influenza B virus | neg (no ct) | neg (no ct) | neg (no ct) | neg (no ct) |
| Influenza B virus | neg (no ct) | neg (no ct) | neg (no ct) | neg (no ct) |
| Influenza B virus | neg (no ct) | neg (no ct) | neg (no ct) | neg (no ct) |
| Influenza B virus | neg (no ct) | neg (no ct) | neg (no ct) | neg (no ct) |
| JC virus | neg (no ct) | neg (no ct) | neg (no ct) | neg (no ct) |
| Parvovirus B19 | neg (no ct) | neg (no ct) | neg (no ct) | neg (no ct) |
| Rhino-/Enterovirus | neg (no ct) | neg (no ct) | neg (no ct) | neg (no ct) |
| Varicella-zoster virus | neg (no ct) | neg (no ct) | neg (no ct) | neg (no ct) |
| **Fungi** |  |  |  |  |
| *Aspergillus* sp. | neg (no ct) | neg (no ct) | neg (no ct) | neg (no ct) |
| *Aspergillus* sp. | neg (no ct) | neg (no ct) | neg (no ct) | neg (no ct) |
| *Pneumocystis jirovecii* | neg (no ct) | neg (no ct) | neg (no ct) | neg (no ct) |
| **Bacteria** |  |  |  |  |
| *Candida albicans* | neg (no ct) | neg (no ct) | neg (no ct) | neg (no ct) |
| *Chlamydia trachomatis, Neisseria gonorrhoeae* | neg (no ct) | neg (no ct) | neg (no ct) | neg (no ct) |
| *Citrobacter koseri* | neg (no ct) | neg (no ct) | neg (no ct) | neg (no ct) |
| *Corynebacterium striatum* | neg (no ct) | neg (no ct) | neg (no ct) | neg (no ct) |
| *Enterobacter cloacae complex* | neg (no ct) | neg (no ct) | neg (no ct) | neg (no ct) |
| *Enterococcus avium* | neg (no ct) | neg (no ct) | neg (no ct) | neg (no ct) |
| *Enterococcus faecalis* | neg (no ct) | neg (no ct) | neg (no ct) | neg (no ct) |
| *Enterococcus faecium* | neg (no ct) | neg (no ct) | neg (no ct) | neg (no ct) |
| *Enterococcus gallinarum* | neg (no ct) | neg (no ct) | neg (no ct) | neg (no ct) |
| *Escherichia coli* | neg (no ct) | neg (no ct) | neg (no ct) | neg (no ct) |
| *Escherichia coli (multiresistant Gram-negative)* | neg (no ct) | neg (no ct) | neg (no ct) | neg (no ct) |
| *Haemophilus influenzae* | neg (no ct) | neg (no ct) | neg (no ct) | neg (no ct) |
| *Klebsiella oxytoca* | neg (no ct) | neg (no ct) | neg (no ct) | neg (no ct) |
| *Klebsiella pneumoniae* | neg (no ct) | neg (no ct) | neg (no ct) | neg (no ct) |
| *Leclercia adecarboxylata* | neg (no ct) | neg (no ct) | neg (no ct) | neg (no ct) |
| *Morganella morganii* | neg (no ct) | neg (no ct) | neg (no ct) | neg (no ct) |
| *Mycoplasma genitalium* | neg (no ct) | neg (no ct) | neg (no ct) | neg (no ct) |
| *Proteus mirabilis* | neg (no ct) | neg (no ct) | neg (no ct) | neg (no ct) |
| *Pseudomonas aeruginosa* | neg (no ct) | neg (no ct) | neg (no ct) | neg (no ct) |
| *Pseudomonas aeruginosa* | neg (no ct) | neg (no ct) | neg (no ct) | neg (no ct) |
| *Serratia marcescens* | neg (no ct) | neg (no ct) | neg (no ct) | neg (no ct) |
| *Staphylococcus aureus* | neg (no ct) | neg (no ct) | neg (no ct) | neg (no ct) |
| *Staphylococcus epidermidis* | neg (no ct) | neg (no ct) | neg (no ct) | neg (no ct) |
| *Stenotrophomonas maltophilia* | neg (no ct) | neg (no ct) | neg (no ct) | neg (no ct) |
| *Streptococcus agalactiae* | neg (no ct) | neg (no ct) | neg (no ct) | neg (no ct) |
| *Streptococcus dysgalactiae* | neg (no ct) | neg (no ct) | neg (no ct) | neg (no ct) |
| *Streptococcus mitis group* | neg (no ct) | neg (no ct) | neg (no ct) | neg (no ct) |
| *Streptococcus pyogenes* | neg (no ct) | neg (no ct) | neg (no ct) | neg (no ct) |
| *Streptococcus sanguinis group* | neg (no ct) | neg (no ct) | neg (no ct) | neg (no ct) |
| *Turicella otitidis* | neg (no ct) | neg (no ct) | neg (no ct) | neg (no ct) |
| *Ureaplasma parvum* | neg (no ct) | neg (no ct) | neg (no ct) | neg (no ct) |
| *Ureaplasma urealyticum* | neg (no ct) | neg (no ct) | neg (no ct) | neg (no ct) |

*(The cross-reactivity study included 32 virus samples, 3 fungal isolates and 32 bacterial isolates. Ct values for influenza B virus positives samples ranged from 16.0 to 25.7.)*

**
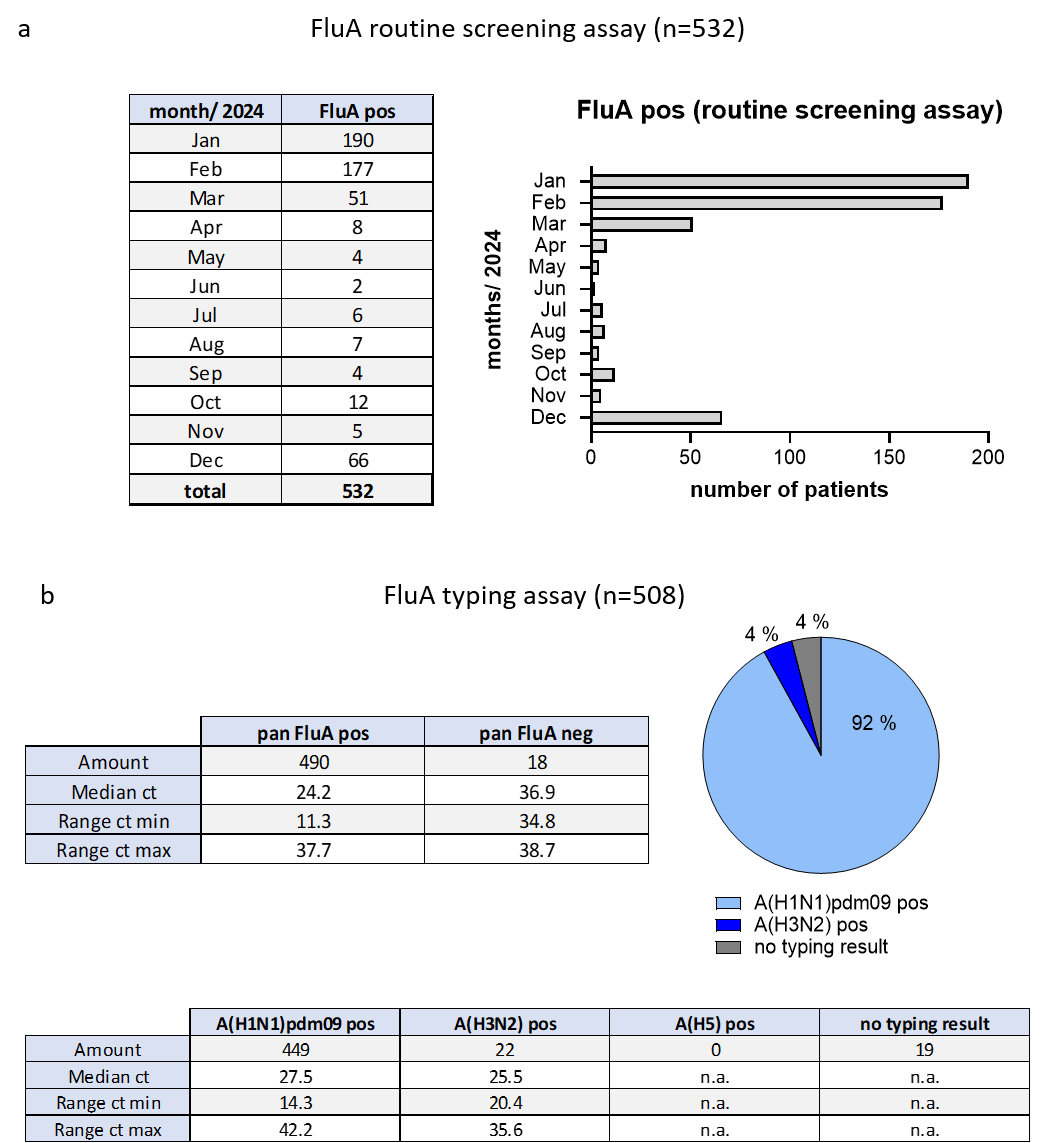
**

***Suppl. figure 1****.* ***Review after one year of use in routine diagnostics.*** ***a****) Number of patients detected with our influenza A (FluA) screening assay in 2024.* ***b****) Number of patients, median ct values and range detected with our* *influenza A subtyping assay in 2024. n.a.= not applicable.*

**Suppl. table 4. Comparison of different influenza A subtyping assays and approaches in terms of time, sensitivity, specificity and cost.**

| **diagnostic method** | **turn around time** | **costs/ per sample** | **remarks** | **reference** |
| --- | --- | --- | --- | --- |
| Our influenza A subtyping panel | ~ 3-4 hours | ~ 10 EUR | - | - |
| Virus isolation | 2-7 days | ~ 70 EUR (isolation) + ~30 EUR (qPCR) | Unit costs for embryonated chicken eggs in EU co-funded monitoring programs | - |
| bioMérieux FilmArray Respiratory Panel 2.1 | ~ 45 min | ~ 190 EUR | Does not include influenza A-H5 | [1] |
| BIOFIRE SPOTFIRE Respiratory/ Sore Throat Panel | ~ 15 min | ~ 190 EUR | Does not include influenza A-H5 | - |
| cobas® eplex respiratory pathogen panel 2 | ~ 100 min | ~ 100 EUR | Does not include influenza A-H5 | [2] |
| Qiagen QIAstat-Dx® Respiratory  SARS-CoV-2 Panel | ~ 60 min | ~ 150 EUR | Does not include influenza A-H5 | [1,3] |
| Seegene Allplex Respiratory Panel 1A Assay | ~ 4-5 hours | ~ 15 EUR | Does not include influenza A-H5 | [1] |

[1] van der Westhuizen C, Newton-Foot M, Nel P. Performance comparison of three commercial multiplex molecular panels for respiratory viruses at a South African academic hospital. Afr J Lab Med 2024;13:2415. https://doi.org/10.4102/ajlm.v13i1.2415.

[2] Phan T, Valeriano P, Boes S, McCullough M, Gribschaw J, Wells A. Evaluation of the ePlex Respiratory pathogen panel 2 to detect viral and bacterial pathogens, including SARS-CoV-2 Omicron in nasopharyngeal swabs. Journal of Clinical Virology Plus 2022;2:100072. https://doi.org/10.1016/j.jcvp.2022.100072.

[3] Boers SA, Melchers WJG, Peters CJA, Toonen M, McHugh MP, Templeton KE, et al. Multicenter Evaluation of QIAstat-Dx Respiratory Panel V2 for Detection of Viral and Bacterial Respiratory Pathogens. Journal of Clinical Microbiology 2020;58:10.1128/jcm.01793-19. https://doi.org/10.1128/jcm.01793-19.
